# Gastrointestinal Helminths of a European Moose Population in Poland

**DOI:** 10.3390/pathogens10040456

**Published:** 2021-04-11

**Authors:** Katarzyna Filip-Hutsch, Michał Czopowicz, Agnieszka Barc, Aleksander W. Demiaszkiewicz

**Affiliations:** 1 Witold Stefański Institute of Parasitology PAS, Twarda 51/55, 00-818 Warsaw, Poland; aldem@twarda.pan.pl; 2 Division of Veterinary Epidemiology and Economics, Institute of Veterinary Medicine, Warsaw University of Life Sciences–SGGW, Nowoursynowska 159c, 02-776 Warsaw, Poland; mczopowicz@gmail.com; 3 Veterinary Diagnostic Laboratory ALAB *bioscience*, Krucza 13, 05-090 Rybie, Poland; agnieszka.barc@alab.com.pl

**Keywords:** *Alces alces*, gastrointestinal tract, liver, helminths, lesions, copromicroscopical analysis

## Abstract

Parasitic infections have a negative impact on the fecundity and survival of wild ruminants, particularly moose; however, despite being more susceptible to parasitic diseases than other wild cervids, they remain poorly examined in this regard. Therefore, the aim of the present study was to identify gastrointestinal and liver helminth species of the moose population in central Europe, assess the factors contributing to infection intensities and examine their impact on moose health. Abomasum, small intestine, caecum and liver samples were collected from 46 moose in Poland and evaluated for helminth parasite fauna and histopathological changes. Additionally, 289 moose fecal samples were analyzed for the presence of eggs, oocysts and larvae of parasites. In total, 19 parasite taxa were identified. The most prevalent were *Mazamastrongylus dagestanica* and *Ostertagia antipini*, which are typical nematodes of moose, together with *Spiculopteragia boehmi* and *O. leptospicularis*, characteristic also of other cervids. Parasite species diversity and abomasal parasitic infection intensity were higher in adult moose than in yearlings and calves. The numbers of histopathological lesions depended on the intensity of parasitic infections, and were most severe in the livers of moose infected with *Parafasciolopsis fasciolaemorpha*. The analysis of fecal samples revealed several regional differences in the levels of parasite eggs, oocysts and larvae shedding. Our findings indicate an accumulation of parasite infections over time in moose, which may be related to high environmental parasite pressure, possibly connected with high moose density and the presence of wetlands; they also serve as the most comprehensive study of moose parasites in central Europe to date.

## 1. Introduction

One of the most important factors affecting wildlife population dynamics is endoparasite infections [1,2]. Gastrointestinal nematodes (GIN) may have a negative impact on the health, fecundity and survival of the host [1,3]. A combination of high intensity of infection and broad parasite species diversity might also significantly impact host health status [4,5]. Ruminants exposed to certain environmental risk factors, such as climate change or high population density, may be particularly susceptible to increasing parasite pressure [2,6,7].

The moose (*Alces alces*), the largest species of cervid, inhabits the boreal forests of central and northern Europe, northern America and Asia [8]. The southernmost area of its distribution runs through Poland, which is also the western border of its European range [9]. The uncontrolled exploitation of the species in the 1980s and 1990s led to a massive population decline and resulted in a ban on moose hunting [9,10]. Since then, the moose population has grown and recently exceeded 28,000 individuals, the majority of whom inhabit eastern and central Poland [11]. 

As heat-sensitive ungulates, moose are particularly susceptible to the effects of global warming [7]. Therefore, being present at high densities, the relatively high-density moose population in Poland presents a unique opportunity to study parasitic infections and host-parasite relationships. Most previous studies concerning moose parasites have been conducted in Scandinavia [3,12]. The only available data from central Europe involve the results of fecal examinations [13,14]. Although such fecal studies may serve as an effective screening method for assessing the parasite fauna of moose, their findings cannot provide a comprehensive view of the intensity of parasitic infections or histopathological changes associated with moose parasitoses. 

The aim of the present study was to identify gastrointestinal and liver helminth species of moose shot, found dead or killed in road accidents in Poland, and to estimate the intensity of infection. It also attempts to identify the risk factors facilitating parasitic infections in moose. 

## 2. Results

### 2.1. Gastrointestinal Tract

#### 2.1.1. Moose Population

The study population comprised 42 moose of three age classes: eight calves (19%), 12 yearlings (29%) and 22 adults (52%). Sex was known for 23 individuals: 14 were female (61%) and nine were male (39%). More moose were from the northeastern (67%) than the central (24%) or eastern region (9%). Neither the age (*p* = 0.985) nor sex (*p* = 0.940) profiles of the animals differed significantly between regions. Body condition was estimated for 18 moose: it was found to be good in three (17%), moderate in nine (50%) and poor in six (33%). Twenty-four moose (57%) had been hunted, 10 (24%) killed in road accidents and eight (19%) had died naturally.

#### 2.1.2. Parasite Species Diversity

Overall, 14 GIN species were identified in the abomasum, including Ostertagiinae, Trichostrongylinae, Haemonchinae and Nematodirinae subfamilies (Table 1). The most prevalent were *Mazamastrongylus dagestanica* and *Ostertagia antipini*, isolated from 100% and over 95% of animals, respectively. The two nematodes together accounted for the vast majority of the total abomasal count in males, with a median interquartile range (IQR) of 95.7% (92.2% to 97.6%). *Ostertagia antipini* predominated in the most intensively infected moose (*p* = 0.032). *Spiculopteragia boehmi*, *O. leptospicularis* together with major morph *O. kolchida* were observed in 66.7–73.8% of moose, depending on the parasite, whereas *O. lyrataeformis* and *O. ostertagi* were present in 12% and 2.4%, respectively. Among the *Trichostrongylus* nematodes, *T. axei* were found in 29% of animals and *T. capricola* in 31%, whereas *T. colubriformis* was isolated only from 2.4% of abomasa. Over 40% of moose were infected with Haemonchinae nematodes such as *Haemonchus* spp. and *Ashworthius sidemi*, followed by *Nematodirella alcidis* in 31% of abomasa. 

All examined moose were infected with GIN, and the number of worms per animal ranged from 60 to virtually 100,000. Severe parasitic infections, i.e., those with more than 40,000 parasites, were observed in 11 of the 42 examined moose (26%). Interestingly, higher counts of nematodes from the Ostertagiinae and Trichostrongylinae subfamilies were observed in moose also infected with parasites from the Haemonchinae subfamily (*p* = 0.004).

**Figure 1:**
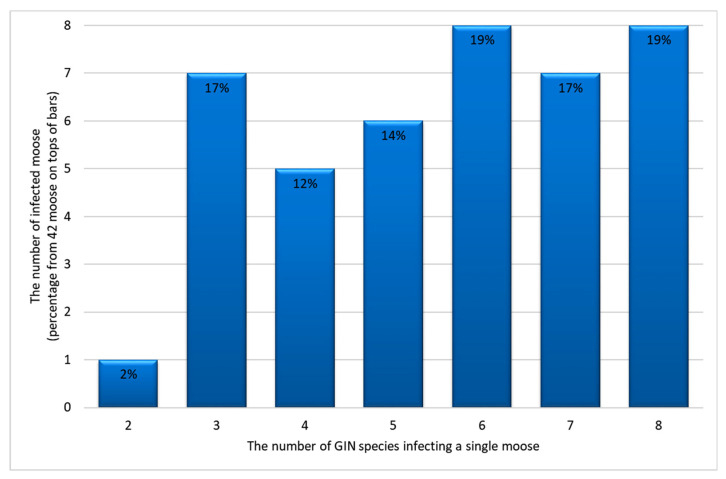
The number (percentage) of moose with abomasal co-infection with two or more gastrointestinal nematode (GIN) species (parasite diversity).

**Figure 2:**
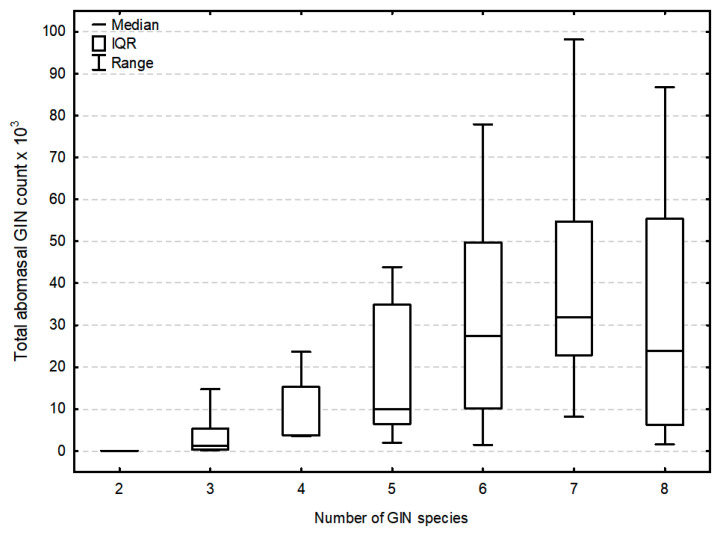
Relationship between the total abomasal gastrointestinal nematode (GIN) count and the number of GIN species infecting a single moose. IQR stands for interquartile range.

Among the 14 GIN identified overall, the number of species infecting individual moose ranged from two to eight, with the median of six (Figure 1). The number of GIN species was significantly positively correlated with the total abomasal GIN counts (Figure 2). 

Thirteen parasite species were isolated from the duodenum (Table 1). Like in the abomasum, gastrointestinal nematodes of the subfamily Ostertagiinae and Trichostrongylinae predominated, with a combined prevalence of 92%, followed by the fluke *Parafasciolopsis fasciolaemorpha* and nematode *N. alcidis*. Flukes from the family Paramphistomidae and *Bunostomum* spp. nematodes were isolated less often, being found in 22% and 14% of intestines, respectively. Unfortunately, no quantitative evaluation of parasitic infections in the small intestine was possible since only fragments of the organ were collected. 

Nematodes from the genus *Trichuris* were isolated from 10 of 12 examined moose caeca (83%). The intensity of infection varied from 10 to 583 nematodes with a median intensity of 41 (Table 1).

#### 2.1.3. Correlations of Parasitic Infections

Generally, adult moose had significantly higher species diversity (*p* = 0.026) and GIN infection intensity (*p* < 0.001) than yearlings and calves, especially for nematodes from the subfamily Ostertagiinae, including *M. dagestanica*, *O. antipini*, *O. leptospicularis* and its major morph *O. kolchida* (Supplementary Table S1). *Ostertagia kolchida* and female nematodes from the subfamily Haemonchinae were also more prevalent in adults (Supplementary Table S2). 

Several regional differences were also observed between the studied regions of Poland. *Spiculopteragia boehmi* was found to be more prevalent in northeastern Poland (Supplementary Table S3). The most severe parasitic infections, e.g., with infection intensity exceeding 40,000 parasites, were significantly more common in eastern Poland; however, only four moose abomasa from this region were included in the study (*p* = 0.002). 

No relationship was observed between parasitic infections and sex, body condition or the cause of death of the examined moose.

#### 2.1.4. Anatomopathological Analysis

The abomasum mucosa was pale pink, with no signs of damage, ulceration or congestion. 

**Figure 3:**
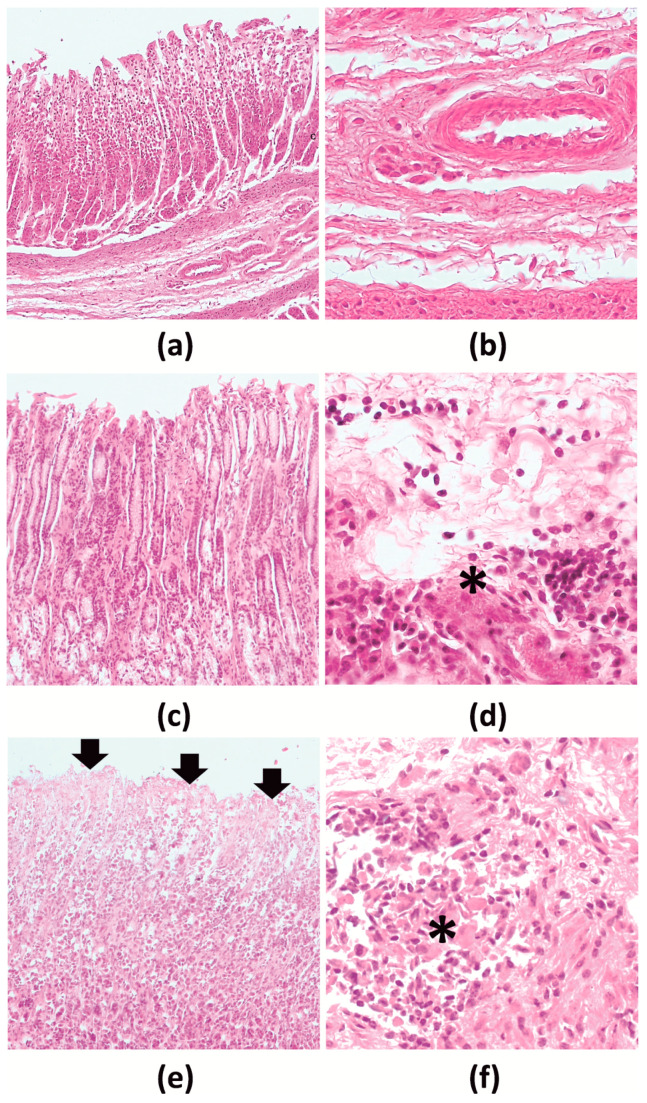
Histopathology of moose abomasa by H-E staining. (**a**,**b**)—normal, (**c**,**d**)—minimal changes, (**e**,**f**)—slight changes; (**a**,**c**,**e**)—Fundic gland region (10× magnification); (**b**,**d**,**f**)—submucosa, inflammatory cell infiltration (40× magnification). *—connective tissue filled with detritus and inflammatory cells; black arrow—exfoliation of epithelium.

Autolytic changes in the four abomasa precluded performing histopathological examination. A number of histopathological lesions were observed in the rest of the examined abomasa: slight exfoliation of the epithelium, small foci of mixed inflammatory cell infiltration which were most prominent in the furrows and submucosa, widened muscularis mucosae, formation of connective tissue caverns filled with detritus and inflammatory cells in the submucosa, submucosa edema and several foci of autolysis/necrosis of the muscularis externa (Figure 3). The intensity of the described changes ranged from minimal to slight, and the level was strongly positively correlated with the degree of parasitic infection (R_s_ = 0.95; *p* < 0.001). The results of histopathological scoring are provided in Supplementary Table S4.

### 2.2. Liver

#### 2.2.1. Moose Population

Livers were collected from 20 moose (nine males and 11 females): 13 in the central region and seven in eastern Poland. Seven of them were calves (35%), three were yearlings (15%), and 10 were adults (50%). No significant difference in the distribution of age classes was found between males and females (*p* = 0.999), nor was there any difference in the distribution of age classes (*p* = 0.170) or sexes (*p* = 0.999) between central and eastern Poland.

#### 2.2.2. Parasite Species and Correlations of Parasitic Infections

*P. fasciolaemorpha* flukes were isolated from 13 moose (65%). The infection intensity ranged from four to 11,150 trematodes, with a median intensity of 300 parasites. Parafasciolopsosis was diagnosed in all animals in the eastern region with a median intensity of 2389 flukes; this value was much higher than in central Poland, with a prevalence of 45% and median infection intensity of 188 parasites. No significant difference in prevalence or intensity of infection was observed between sexes (*p* = 0.642 and *p* = 0.524, respectively) or age classes (*p* = 0.350 and *p* = 0.171, respectively). However, a slight tendency was observed for a higher prevalence in adult animals (8 of 10, 80%) compared to yearlings (2 of 3, 67%) and calves (3 of 7, 43%).

#### 2.2.3. Anatomopathological Analysis

Livers of moose infected with *P. fasciolaemorpha* were firm and enlarged, with rounded edges. On the surface, rounded cream-colored spots with a diameter ranging from 1 to 3 cm were visible. The lobular pattern was visible on the cross-section together with thickening of the bile ducts and multiple cavities filled with flukes, their eggs and cellular detritus. 

**Figure 4:**
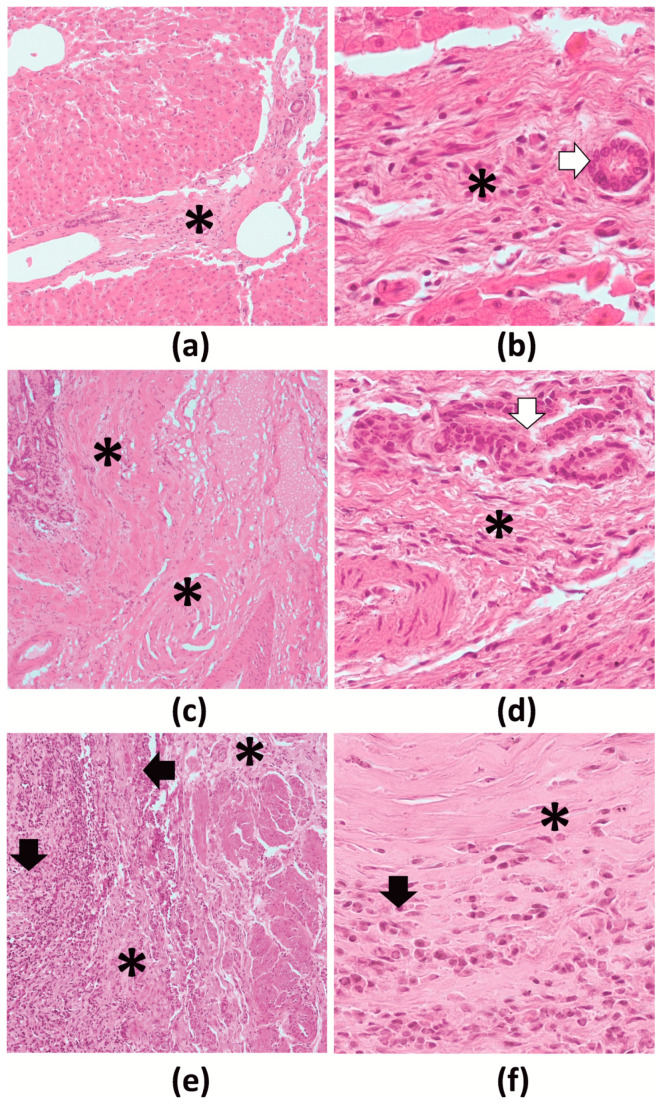
Histopathology of moose livers in H-E staining. (**a**,**b**)—slight changes, (**c**,**d**)—moderate changes, (**e**,**f**)—severe changes; (**a**,**c**,**e**)—bands of fibrous tissue (10× magnification); (**b**,**d**,**f**)—portal region (40× magnification). *—connective tissue hyperplasia; black arrow—mononuclear infiltration; white arrow—biliary epithelium.

Histopathological lesions were found in all liver zones: parenchyma, central vein regions and portal areas. A number of lesions were observed: extensive liver fibrosis ranging from incomplete septal cirrhosis up to complete cirrhosis, formation of layered capsules of connective tissue in the liver parenchyma filled with exfoliated epithelium and fluid, granulation tissue formation foci, dilation and disruption of the bile ducts with local hyperplasia, foci of hepatocyte necrosis, regenerative nodules, mixed inflammatory cell infiltration (most prominent in the portal areas, but also visible multifocally in the parenchyma), teleangiectasia (mainly of the sinusoids but also of the veins and arteries), as well as hypertrophy of the tunica media with hyperplasia of the tunica intima in several arteries (Figure 4). A strong positive correlation was observed between the intensity of parasitic infection and the severity of histopathological lesions in the liver (R_s_ = 0.95; *p* < 0.001). The results of histopathological scoring are provided in Supplementary Table S5.

### 2.3. Fecal Examination

The examination of 289 fecal samples revealed the presence of eggs, oocysts or larvae of 12 parasite species or groups, including gastrointestinal nematodes from the Trichostrongylidae family and *N. alcidis*, nematodes from the genera *Aonchotheca* and *Trichuris*, tapeworms from the genus *Moniezia*, flukes *P. fasciolaemorpha* and flukes from the family Paramphistomidae (Table 2). The most prevalent were Trichostrongylidae eggs, found in 99.0% of samples, followed by *Trichuris* spp. (68.9%), *P. fasciolaemorpha* (64.4%) and *N. alcidis* (61.2%). Eggs from the family Paramphistomidae (29.4%) were less common. The prevalence of other parasites did not exceed 10%. 

The most intensive excretion of eggs or larvae was observed for *Moniezia* spp., followed by the Trichostrongylidae, and *Trichuris* spp.

Some parasitic species demonstrated regional differences regarding excretion. More prevalent *N. alcidis* and *P. fasciolaemorpha* infection was observed in the eastern region than the northeastern and central regions. Eggs of the fluke from the family Paramphistomidae were excreted most often by moose from central Poland. Lower levels of Trichostrongylidae and *Trichuris* spp. eggs excretion were observed in northeastern regions (Supplementary Table S6).

## 3. Discussion

The examination of the moose alimentary tract revealed the presence of 14 GIN species in the abomasum and 13 parasitic species in the duodenum. In general, abomasal and duodenal species composition was found to be similar to those revealed by previous studies in northern Europe [3,12]. The most prevalent were *M. dagestanica* and *O. antipini*, together with its less prevalent major morph *O. lyrataeformis*; these are considered the typical abomasal nematodes of moose [3,12], being phylogenetically linked with the host and being spread throughout the entire range of moose distribution [15]. This also applies to other specific moose parasites, such as the intestinal nematode *N. alcidis*, which in our studies was also accidentally isolated from the abomasum. Typical nematodes of other cervids, including *S. boehmi*, *O. leptospicularis* and its major morph *O. kolchida*, were also very common, followed by the less prevalent *O. ostertagi*: a common parasite of bovids. This may be evidence of the sympatric occurrence of moose and other wild ruminants in these regions, which favors the inter-species transmission of some gastrointestinal parasitoses [16]; for example, at common watering places [17]. 

Nematodes from the subfamily Haemonchinae, including *Haemonchus* spp. and *Ashworthius sidemi*, were significantly more prevalent in moose in Poland than in Scandinavia [3,12]. Both haematophagus nematodes may be the cause of severe anemia and considerable morbidity in domestic and wild ruminants [18,19]. Despite being abomasal nematodes, *Haemonchus* spp. were probably accidentally isolated from the duodenum. Of the two, *Ashworthius sidemi* merits particularly close monitoring, as it is a new parasite in central Europe [20], and the moose population includes a significant percentage of migratory individuals [7]. In the present study, the presence of Haemonchinae infection appeared to predispose the host to higher counts of other abomasal parasites, and thus could worsen the course of parasitic infections, as previously observed in other moose parasites [21,22]. However, Haemonchinae nematodes, having wide-ranging immunomodulatory capacities [23], may also inhibit the occurrence of other nematodes under some conditions [24]. 

Adult animals were more likely to show mixed infections by GIN species than yearlings or calves. In addition, the adult moose also displayed the highest intensity of infection, especially for the most common GIN species—*M. dagestanica*, *O. antipini*, *O. leptospicularis* and its major morph *O. kolchida*. Similar age-related trends have also been observed in moose and red deer in northern Europe [12,25]. 

Moose typically show extremely high intensities of GIN infection [10,12]. Among 42 examined individuals, eleven (26%) showed total abomasal counts exceeding an extreme intensity of 40,000 nematodes, with the highest intensity of infection approaching 100,000 GIN. Similarly, while high parasite burdens were observed in dead or debilitated moose in Sweden, significantly less GIN were found in healthy animals culled during hunting season in Norway. In the present study, neither prevalence nor total parasitic counts differed between the animals intentionally culled and those found dead. Grandi et al. [12] proposed that the severe intensity of parasitic infections observed in moose may be connected with the high population density in Poland, as parasite environmental pressure is believed to increase together with population density [11]. 

Severe parasite burdens can negatively influence certain aspects of animal health status, including body mass and fecundity [1,26,27]. In moose in Poland, parasite species diversity increases together with total GIN counts (Figure 2), indicating that the most infected individuals might be more susceptible to new parasitoses. While abomasal loads exceeding 8,000 parasites are believed to be pathogenic for cervids [28], over half the moose examined in the present study exceeded this level. It must be considered, though, that no relationship was observed between the condition of the moose and the intensity of parasitic infection; however, as body condition could not be estimated in all animals, this observation needs stronger confirmation. No significant histopathological changes which precluded regular physiological function were observed in the abomasa; however, to fully determine the damage of the abomasal wall, measurements of serum pepsinogen should be performed [29]. 

Nematodes of the genus *Trichuris* were isolated from over 80% of examined moose caeca and from over 70% of moose fecal samples; these figures are significantly higher than recorded in previous studies [3,12]. Nematodes of the genus *Trichuris* are considered particularly pathogenic for moose [30], contributing to the emaciation of infected individuals during winter [3]; however, again, no such relationship was observed in the present study. Infection with *Trichuris* spp. should be recorded as, according to Davidson et al. [3], it might be influenced by regional or seasonal differences. 

In contrast, the histopathological examinations revealed severe pathological lesions in the livers of animals infected with the fluke *P. fasciolaemorpha*. Extensive fibrosis was observed, together with changes in the blood vessels, which possibly resulted in restricted blood circulation. In the most heavily infected moose, liver parenchyma had been replaced by connective tissue to a degree that substantially compromised regular organ physiological function. The likelihood of liver fibrosis appeared to increase together with the number of trematodes in the organ, as previously noted by Marcos et al. [31]. Such extensive fibrosis, and thus significant disturbances in hepatic blood flow and portal circulation, may be the cause of other clinical ailments, including growth and fertility disorders [32]. Therefore, parafasciolopsosis should be considered as one of the most dangerous types of moose parasitosis. 

*Parafasciolopsis fasciolaemorpha* was isolated from 65% of the examined moose livers and over 66% of small intestines in the present study, which is slightly less than in previous studies based on fecal analysis [13,14]. Its presence appears to be limited to central and eastern Europe and its occurrence varies depending on the presence of wetlands inhabited by water snails, these being the typical intermediate hosts of the trematode [13]. Thus, in our study, infection was mostly restricted to watershed areas of eastern Poland. The level of trematode infection in the moose in the Kampinos forest, central Poland, was lower, possibly due to the presence of sandy dunes which can reduce water snail survival and thus parafasciolopsosis transmission. Fecal examination revealed a similar tendency for a higher prevalence of *P. fasciolaemorpha* eggs in eastern Poland than the other studied areas. 

The analysis of 289 fecal samples revealed the presence of eggs, oocysts and larvae from 12 parasite species or groups, all previously reported in moose [3,10,13]. The eggs of gastrointestinal nematodes from the family Trichostrongylidae were present in virtually all examined samples, which corresponds with the post-mortem examinations. All the other most prevalent species were parasites of the digestive system; these were also isolated in the post-mortem analysis: *Trichuris* spp., *P. fasciolaemorpha* and *N. alcidis*. 

The occurrence and intensity of parasitic infections are influenced by a range of seasonal and regional factors, which were also observed in our study. The excretion of Trichostrongylidae eggs, *Trichuris* spp. and *P. fasciolaemorpha* was significantly lower in northeastern Poland than other studied areas, while *N. alcidis* and *P. fascioalemorpha* eggs were considerably more present in eastern Poland. It is possible that, in northeastern regions, the transmission of some parasitic species is inhibited by low annual temperatures [13]. Eggs of the fluke *Paramphistomum* spp., typical for many species of ruminants [10], were excreted more often in moose in central Poland; this may also be an evidence of inter-species parasitic transmission. 

Generally, the prevalence of other gastrointestinal parasites, including the eggs of *Aonchotheca* spp. and *Moniezia* spp., was low and did not exceed 13%, which is in line with studies on moose in Scandinavia [3,12]. 

On the other hand, these values are significantly lower than those observed in a previous study based on molecular examination of moose feces [14]. As molecular evaluation is more sensitive, the results obtained by fecal egg counts based on traditional coproscopic examination should be treated with caution [3,14].

Generally, parasitic infections and host-parasite relationships might be influenced by a range of ecological and biological factors [2], which were also observed in the present study. In ruminant populations, a high herd density favors contact between individuals and thus greater transmission of parasites. Some parasite species might be introduced to moose herds by the presence of other wild animals or domestic ruminants in the environment [16,17]. Moreover, helminth development, transmission and survival in the environment strongly depends on climate and season, especially abiotic factors, to which the life cycles of specific parasites are adapted [13]. Access to water sources enables the survival of intermediate hosts or some larval forms of parasites and thus favors the transmission of parasitoses, e.g., parafasciolopsosis [13]. Individual factors, like immunological status or age of the host, might also favor or decrease the risk of parasitic infections [2,14], e.g., tapeworms from the genus *Moniezia* are more prevalent in young animals, whereas gastrointestinal nematodes dominate in adult moose. During the present study, no differences in parasitic loads were observed between moose sexes, despite such relationships being observed previously [3,13,14]. Therefore, when analyzing parasitic infections in the moose population, a number of factors should be taken into consideration, especially in the face of global warming and, with it, increasing parasite pressure.

## 4. Materials and Methods

### 4.1. Study Area

The study was conducted in six locations in northeastern, eastern and central Poland within the moose core area, inhabited by about 70% of the population [33]. In all six study locations, two other cervid species, namely red deer and roe deer, were present, with roe deer predominating [34].

The four study areas, namely the Biebrza marshland (53°24′25″ N, 22°47′43″ E), the Knyszyn Forest (53°10′35″ N, 23°48′47″ E), the Augustów Forest (53°58′46″ N, 23°17′23″ E) and the Białowieża Primeval Forest, are located in Podlaskie Voivodeship in northeastern Poland. The region has a continental climate, with warm summers and long winters. The mean annual air temperature is 7 °C. Precipitation varies around 550 mm and the vegetation period lasts 200 to 210 days. However, the environment of the Podlaskie Voivodeship is extremely diverse, ranging from the Biebrza marshland, the largest watershed area in Europe, to the vast and primary forests of Białowieża, Augustów and Knyszyn. 

West Polesie (51°23′37″ N, 23°11′41″ E), eastern Poland, is located on the border of Belarus and Ukraine, in the area of the Bug and Wieprz rivers. The climate is typical of Great Valleys with some continental features, i.e., a long summer and winter. The mean annual air temperature is 7.3 °C. Precipitation varies from 400 mm to 850 mm and most of the rain falls in the summer [35]. It demonstrates a wide heterogeneity of habitats, with peat bogs, meadow complexes and mid-forest turf lands occupied mostly by pine and alder.

The Kampinos Forest (52°18′00″ N, 20°49′48″ E) is located in the Mazovian Lowland in central Poland and covers a part of the ancient valley of the Vistula basin. It lies in the temperate climate zone and is exposed to transitional marine and continental influences. The mean annual temperature is 7.7 °C and the precipitation is 547 mm [36]. The forest is a combination of sandy dunes and marshes, with dense pine and spruce afforestation.

### 4.2. Material Collection and Post-Mortem Examination

Post-mortem examinations were carried out on 46 moose cadavers in the area of northeastern, eastern and central Poland. Twenty-four animals with no signs of diseases, injuries or other disorders were culled by hunters in 2010 in northeastern Poland in accordance with the decision of the Ministry of Environment: DL.gL-6713-5/45392/10/PJ approved by the Local Bioethical Committee in Białystok (permission: 48/2010). The other 22 moose were killed in road accidents or found dead in the years 2015–2020. 

The post-mortem examinations were performed in the field according to standard necropsy techniques and parasitological procedures [37], no more than two days after the animals’ death. The moose were divided into three age classes based on tooth development and wear [38]: calves (two to eight months old), yearlings (one to two years old) and adults (more than two years old). Sex was specified for 23 animals. Body condition was determined by subjective field assessment of pericardiac and perirenal fat reserves in 18 animals [39].

The abomasum, duodenum, caecum and liver were collected during the moose dissections, then immediately transported to the laboratory and examined or frozen at −20 °C until further analyses could be done. However, due to the condition of the carcass, it was not always possible to collect all the parts, and the number of examined organs differed between animals. Samples of the organs were preserved during the dissection of moose in 10% buffered formalin for further histopathological analysis.

#### 4.2.1. Gastrointestinal Tract

In total, 42 abomasa were collected from 28 moose in northeastern Poland, 10 in central and four in eastern Poland. The abomasum was cut along the greater curvature and rinsed in tap water. The content of the abomasa was sedimented and 1/10 volume of the sediment was examined under a stereoscopic microscope (PZO, Poland) at 40× magnification. 

Isolated nematodes were differentiated into males and females, preserved in a 70% alcohol and counted. The counts were then multiplied to yield estimated total counts (the number of parasites × 10) for further analysis. All recovered parasites were identified on the basis of morphometrical features [37,40,41]. 

The male nematodes were mounted in a lactophenol solution in a dorsal position under a Jenaval microscope (Carl Zeiss Jena, Jena, Germany) at 20–100× magnification for further species identification [37,40]. All measurements were performed taking into consideration the Ostertagiinae male polymorphism, which affected the morphology and length of the spicules and the genital cone [41]. However, different morphotypes were considered as a different species for further analysis. Females were identified only to the subfamily level.

The duodenum was collected from 36 moose and the caecum from 12; by region, 23 duodena and three caeca in northeastern Poland, nine and 10 in central Poland, and four and two in eastern Poland. The duodenum and caecum were cut and the content was sedimented as described above. Following this, the total content of the duodenum or caecum was examined under a stereoscopic microscope (PZO, Warsaw, Poland) at 40× magnification and 10× magnification respectively. After isolation, the parasites were preserved in 70% alcohol, counted and identified to the species or genus level on the basis of morphometrical features, as described above [41]. 

#### 4.2.2. Liver

Twenty livers were collected: seven moose in eastern Poland and 13 in central Poland. The livers were cut into small pieces, compressed and rinsed in a tap water so that the flukes and their eggs could leave the bile ducts. The decantated liver sediment was examined under a stereoscopic microscope (PZO, Poland) at 10× magnification. The isolated flukes were identified on the basis of morphometrical features [42,43] and counted. 

### 4.3. Anatomopathological Analysis

Macroscopic examination of liver was performed during the dissections of moose whereas anatomopathological investigation of abomasum was performed immediately after transport to the laboratory. The size, shape, surface and cross-section of the liver as well as the surface of the abomasal mucosa were assessed. 

Histopathological assessment was performed of 20 livers and abomasa. Seven and two samples of liver and abomasum, respectively, were collected from uninfected or poorly infected moose; these were considered as controls. 

Liver samples were collected from the areas of visible gross lesions, or from the edge and center of the right and left lobes in case of normal-looking organs. All parts of the abomasal wall were cut from the pyloric area. Samples were fixed in 10% buffered formalin during parasitological dissection. 

Under laboratory conditions, tissue sections were macroscopically examined and then dehydrated in graded ethanol and xylene baths. The dehydrated sections (measuring 3–4 µm) were then embedded in paraffin wax. The sections were stained with haematoxylin and eosin (H-E). Microscopic evaluation was performed at 10× and 40× magnification and the sample was photographed. The structure of the liver and abomasum wall tissue were examined using an Axiolab A5 light microscope with Axiocam 208 color and ZEN 3.0 software (Zeiss, Jena, Germany).

The detailed assessment of regressive changes such as necrosis, degeneration and atrophy were not included in the histopathological analysis due to developing autolysis. The histopathological evaluation was carried out in two stages. In the first part, histopathological changes were identified and those associated with autolysis were rejected. In the second part, a scoring assessment of selected pathological processes was performed. 

The liver scoring criteria were as follows: cirrhosis/liver fibrosis, inflammatory infiltrates, lobule architectural disturbance and formation of connective tissue capsules. In the abomasum, scoring included exfoliation of the mucosa epithelium, inflammatory infiltrates in the mucosa, inflammatory infiltrates in the submucosa, tunica mucosa thickening and cavern formation. The changes were assessed on the following scale: 0-no lesions, 1-minimal, 2-mild, 3-moderate, 4-severe.

### 4.4. Fecal Analysis

A total of 289 moose fecal samples were collected from December 2017 to February 2018: 148 samples in West Polesie, 101 samples in Kampinos Forest, 30 in Biebrza marshland and 10 in Augustów Forest. The sampling strategy included both temporal and spatial stratification to avoid pseudoreplication, i.e., no more than one or two fecal samples were collected from a given area in the same time. The samples were transported at 4 °C in anaerobic conditions and examined in the laboratory within five days after collection. 

Three grams of each sample were examined for gastrointestinal parasite eggs and oocysts using direct flotation in a sucrose solution followed by centrifugation [44]. The presence of fluke eggs was determined by decantation in a tap water according to Żarnowski and Josztowa [45], and lung nematode larvae were detected using the Baermann technique [46]. The samples were observed under a stereoscopic microscope (PZO, Poland) at 40× magnification. Eggs, oocysts and larvae were identified to the family, genus or species level on the basis of morphology [42,43,47,48].

Two parameters were used to present the levels of parasitic infections: prevalence, defined as the percentage of moose feces in which parasite eggs were detected at least once to the number of examined samples, and intensity, defined as the number of eggs in 1 g of feces (EPG).

### 4.5. Statistical Analysis

Categorical variables were presented as count and percentage and compared between groups using the Pearson’s χ2 test; if the expected count in any cell of the two-by-two contingency table was ≤5, Fisher’s exact test was used. The Wilson score method was used to determine the 95% confidence intervals (CI 95%) for proportions [49]. Numerical variables were expressed as median, interquartile range (IQR) and range, and compared between groups using the Mann–Whitney U-test (two groups) or Kruskal–Wallis H-test (more than two groups). Correlations of numerical and ordinal variables were assessed with Spearman’s rank correlation coefficient (R_s_). Correlation was considered as strong when R_s_ was >0.7 or <−0.7 [50]. All analyses were univariable and all statistical tests were two-tailed. The significance level (α) was set at 0.05. Statistical analysis was performed in TIBCO Statistica 13.3.0 (TIBCO Software Inc., Palo Alto, CA, USA).

## 5. Conclusions

In total, 19 species of gastrointestinal helminths were identified in the studied moose on the basis of both post-mortem and fecal examination; however, these numbers could have been underestimated since only parts of small and large intestine were examined on the presence of parasites and some helminths eggs in the fecal analysis were identified only to the genus level. The higher species diversity and abomasal parasite burdens observed in adult animals, which suggests a gradual accumulation of parasitic infections and high environmental parasitic pressure, especially related to the presence of wetlands. Increasing population density could favor parasite transmission and contamination of the environment; this is especially true for the fluke *P. fasciolaemorpha*, which should be considered the most pathogenic parasite for moose in the present study. 

Further studies are needed to fully assess the changes related to extremely intensive parasitic infections in moose and to determine their impact on the health status of individual moose. It is important to note that the damage caused by endoparasites is often insidious, and the effects may only become apparent in the long term [51]. Therefore, there is a strong need to monitor parasitic infections in the unique moose population of central Europe.

## Figures and Tables

**Table 1:** Prevalence and abundance of helminth parasites recovered from the gastrointestinal tract and liver of examined moose.

Parasitic Species	No. of Infected Moose	Prevalence (CI 95%)	Parasite Count Median (Range)
Abomasum (*n* = 42)			
*Mazamastrongylus dagestanica*	42	100 (91.6–100)	1410 (5–28,900)
*Ostertagia antipini* ^a^	40	95.2 (84.2–98.7)	2920 (1–33,550)
*Ostertagia leptospicularis* ^b^	29	69.0 (54.0–80.9)	60 (2–585)
*Ostertagia kolchida*	28	66.7 (51.6–79.0)	50 (1–800)
*Ostertagia lyrataeformis*	5	11.9 (5.2–25.0)	40 (10–80)
*Ostertagia ostertagi*	1	2.4 (0.4–12.3)	10
*Trichostrongylus axei*	12	28.6 (17.2–43.6)	20 (10–3300)
*Trichostrongylus colubriformis*	1	2.4 (0.4–12.3)	1
*Trichostrongylus capricola*	13	31.0 (19.1–46.0)	10 (1–150)
*Spiculopteragia boehmi*	31	73.8 (58.9–84.7)	110 (4–890)
Trichostrongylinae& Ostertagiinae ♀ ♀	42	100 (91.6–100)	7760 (30–61,900)
**Subfamily: Trichostrongylinae & Ostertagiinae**	42	100 (91.6–100)	13,195 (60–98,150)
*Haemonchus* spp.	11	26.2 (15.3–41.1)	18 (1–260)
*Ashworthius sidemi*	8	19.0 (10.0–33.3)	13 (1–776)
Haemonchinae ♀ ♀	15	35.7 (23.0–50.8)	20 (1–503)
**Subfamily: Haemonchinae**	17	40.5 (27.0–55.5)	50 (2–1300)
*Nematodirella alcidis*	13	31.0 (19.1–46.0)	60 (1–200)
**Total abomasal GIN**	42	100 (91.6–100)	13,333 (60–98,159)
Duodenum (*n* = 35)			
*Mazamastrongylus dagestanica*	24	66.7 (50.3–79.8)	3 (1–197)
*Ostertagia antipini* ^a^	21	58.3 (42.2–72.9)	4 (1–127)
*Ostertagia leptospicularis* ^b^	3	8.3 (2.9–21.8)	2, 3, 3
*Ostertagia kolchida*	3	8.3 (2.9–21.8)	1, 1, 6
*Ostertagia lyrataeformis*	1	2.8 (0.5–14.2)	1
*Trichostrongylus axei*	18	50.0 (34.5–65.5)	7 (1–17)
*Trichostrongylus capricola*	24	66.7 (50.3–79.8)	5 (1–24)
*Spiculopteragia boehmi*	7	19.4 (9.8–35.0)	3 (1–17)
Trichostrongylinae& Ostertagiinae ♀ ♀	33	91.7 (78.2–97.1)	23 (2–791)
**Subfamily: Trichostrongylinae & Ostertagiinae**	33	91.7 (78.2–97.1)	45 (2–1002)
*Haemonchus* spp.	2	5.6 (1.5–18.1)	1, 1
*Nematodirella alcidis*	21	58.3 (42.2–72.9)	4 (1–208)
*Bunostomum* spp.	5	13.9 (6.1–28.7)	8 (2–269)
*Parafasciolopsis fasciolaemorpha*	24	66.7 (50.3–79.8)	26 (1–1538)
Paramphistomidae	8	22.2 (11.7–38.1)	3 (1–71)
Caecum (*n* = 12)			
*Trichuris* spp.	10	83.3 (55.2–95.3)	41 (10–583)
Liver (*n* = 20)			
*Parafasciolopsis fasciolaemorpha*	13	65.0 (43.3–81.9)	300 (4–11,150)

^a^ Minor morph of *O. lyrataeformis*. ^b^ Minor morph of *O. kolchida*. ♀ ♀ Female nematodes. *n*—number of examined individuals. CI 95%—the upper and lower bounds of the confidence interval.

**Table 2:** Overall prevalence and intensity of parasitic infections in 289 fecal samples from moose.

Parasite	No. of Infected Moose	Prevalence (CI 95%)	Intensity [EPG] Median (Range)
Trichostrongylidae	286	99.0 (97.0–99.6)	7 (<1–1076)
*Nematodirella alcidis*	177	61.2 (55.5–66.7)	1 (<1–30)
*Aonchotheca* sp.	37	12.8 (9.4–17.1)	<1 (<1–4)
*Trichuris* spp.	199	68.9 (63.3–73.9)	4 (<1–978)
*Moniezia* spp.	27	9.3 (6.5–13.3)	18 (<1–217)
*Parafasciolopsis fasciolaemorpha*	186	64.4 (58.7–69.7)	1 (<1–872)
Paramphistomidae	85	29.4 (24.5–34.9)	1 (<1–19)

CI 95%—the upper and lower bounds of the confidence interval, EPG—eggs per gram.

## Data Availability

The data that support the findings of this study are available from the corresponding author, upon reasonable request.

## Supplementary Materials

The following are available online at https://www.mdpi.com/article/10.3390/pathogens10040456/s1. Supplementary Table S1: Prevalence (95% confidence interval) of parasitic infections in moose abomasa according to age class. Calves and yearlings were merged into one category. Supplementary Table S2: Intensity of gastrointestinal nematode (GIN) infections presented as the median and range in moose abomasum according to age class. Calves and yearlings were merged into one category, and only these parasites which were present in both age classes were included. Supplementary Table S3: Prevalence of gastrointestinal nematode (GIN) infections in moose abomasum in the studied regions of Poland. Supplementary Table S4: Prevalence and infection intensity of eggs, oocysts and larvae of parasites in the feces according to the studied region of Poland. The arrows indicate how the prevalence and number of excreted eggs, oocysts and larvae differed. Supplementary Table S5: Histopathological scoring of the moose liver. Supplementary Table S6: Prevalence and infection intensity of eggs, oocysts and larvae of parasites in the feces according to the studied region of Poland. The arrows indicate how the prevalence and number of excreted eggs, oocysts and larvae differed.
