# Supplementary material for: Gastrointestinal Helminths of a European Moose Population in Poland
Source: Pathogens. 2021 Apr 11;10(4):456. doi: 10.3390/pathogens10040456 (PMC8070461; doi:10.3390/pathogens10040456)
Supplement: Supplementary file 1 [file pathogens-10-00456-s001.pdf]

Supplementary Table S1. Prevalence (95% confidence interval) of parasitic infections in moose abomasa according to age class. Calves and yearlings were merged into one category.

Parasite	Calves / Yearlings (*n* = 20)		Adults (*n* = 22)		
	n	% (CI 95%)	*n*	% (CI 95%)	*p*-value
Mazamastrongylusdagestanica	20	100 (83.9–100)	22	100 (85.1–100)	>0.999
Ostertagia antipini[a]	18	90.0 (69.9–97.2)	22	100 (85.1–100)	0.221
Ostertagia leptospicularis[b]	11	55.0 (34.2–74.2)	18	81.8 (61.5–92.7)	0.060
Ostertagia kolchida	9	45.0 (25. –65.8)	19	86.4 (66.7–95.3) ↑	0.005*
Ostertagia lyrataeformis	2	10.0 (2.8–30.1)	3	13.6 (4.7–33.3)	>0.999
Ostertagia ostertagi	0	0 (0–16.1)	1	4.5 (0.8–21.8)	>0.999
Trichostrongylus axei	5	25.0 (11.2–46.9)	7	31.8 (16.4–52.7)	0.625
Trichostrongylus colubriformis	1	5.0 (0.9–23.6)	0	0 (0–14.9)	0.476
Trichostrongylus capricola	7	35.0 (18.1–56.7)	6	27.3 (13.2–48.2)	0.589
Spiculopteragia boehmi	14	70.0 (48.1–85.5)	17	77.3 (56.6–89.9)	0.592
Trichostrongylinae& Ostertagiinae ♀♀	20	100 (83.9–100)	22	100 (85.1–100)	>0.999
**Subfamily**: **Trichostrongylinae& Ostertagiinae**	20	100 (83.9–100)	22	100 (85.1–100)	>0.999
Haemonchus spp.	3	15.0 (5.2–36.0)	8	36.4 (19.7–57.0)	0.116
Ashworthius sidemi	2	10.0 (2.8–30.1)	6	27.3 (13.2–48.2)	0.243
Haemonchinae♀♀	3	15.0 (5.2–36.0)	12	54.6 (34.7–73.1) ↑	0.008*
**Subfamily: Haemonchinae**	5	25.0 (11.2–46.9)	12	54.6 (34.7–73.1)	0.051
Nematodirella alcidis	6	30.0 (14.5–51.9)	7	31.8 (16.4–52.7)	0.899

[a] Minor morph of *O. lyrataeformis*

[b] Minor morph of *O. kolchida*

♀♀ – female nematodes

*Statistically significant

n – number of infected individuals

CI 95% – the upper and lower bounds of the confidence interval

Supplementary Table S2. Intensity of gastrointestinal nematode (GIN) infections presented as the median and range in moose abomasum according to age class. Calves and yearlings were merged into one category and only these parasites which were present in both age classes were included.

Parasite	Calves / Yearlings (n = 20)		Adults (n = 22)		p
	n	Median (range)	n	Median (range)	
Mazamastrongylusdagestanica	20	997 (5–5240)	22	2415 (330–28 900)	0.006*
Ostertagia antipini[a]	18	271 (1–9240)	22	7780 (30–33 550)	<0.001*
Ostertagia leptospicularis[b]	11	40 (2–140)	18	110 (20–585)	0.020*
Ostertagia kolchida	9	30 (1–80)	19	60 (2–800)	0.040*
Trichostrongylus axei	5	10 (10–210)	7	30 (10–3300)	0.129
Trichostrongylus capricola	7	10 (1–100)	6	20 (3–150)	0.466
Spiculopteragiaboehmi	14	80 (4–890)	17	120 (10–359)	0.311
Trichostrongylinae& Ostertagiinae ♀♀	20	2900 (30–27 400)	22	20 015 (780–61 900)	<0.001*
**Subfamily: Trichostrongylinae& Ostertagiinae**	20	4530 (60–40 960)	22	32 255 (1430–98 150)	<0.001*
Haemonchus spp.	3	10 (10–70)	8	20 (1–260)	0.918
Haemonchinae♀♀	3	30 (20–40)	12	18 (1–503)	0.664
**Subfamily: Haemonchinae**	5	50 (3–100)	12	55 (2–1300)	0.562
Nematodirella alcidis	6	25 (2–90)	7	80 (1–200)	0.431
**Total abomasal GIN count**	20	4530 (60–40 963)	22	32 273 (1432–98 159)	<0.001*

[a] Minor morph of *O. lyrataeformis*
[b] Minor morph of *O. kolchida*
♀♀ – female nematodes
*Statistically significant
n – number of infected individuals

Supplementary Table S3. Prevalence of gastrointestinal nematode (GIN) infections in moose abomasum in the studied regions of Poland.

Parasite	Central Poland (*n* = 10)		Eastern Poland (*n* = 4)		Northeastern Poland (*n* = 28)		
	n	%	*n*	%	*n*	%	*p*
Mazamastrongylusdagestanica	10	100	4	100	28	100	>0.999
Ostertagia antipini[a]	9	90.0	4	100	27	96.4	0.640
Ostertagia leptospicularis[b]	5	50.0	4	100	20	71.4	0.168
Ostertagia kolchida	7	70.0	4	100	17	60.7	0.287
Ostertagia lyrataeformis	3	30.0	0	0	2	7.1	0.118
Ostertagia ostertagi	0	0	0	0	1	3.6	0.774
Trichostrongylus axei	0	0	2	50.0	10	35.7	0.061
Trichostrongylus colubriformis	0	0	0	0	1	3.6	0.774
Trichostrongylus capricola	2	20.0	1	25.0	10	35.7	0.630
Spiculopteragiaboehmi	3	30.0↓	4	100	24	85.7	0.001*
Trichostrongylinae& Ostertagiinae ♀ ♀	10	100	4	100	28	100	>0.999
**Subfamily: Trichostrongylinae& Ostertagiinae**	10	100	4	100	28	100	>0.999
Haemonchus spp.	2	20.0	1	25.0	8	28.6	0.868
Ashworthiussidemi	0	0	2	50.0	6	21.4	0.085
Haemonchinae♀ ♀	2	20.0	1	25.0	12	42.9	0.387
**Subfamily: Haemonchinae**	2	20.0	2	50.0	13	46.4	0.316
Nematodirella alcidis	2	20.0	1	25.0	10	35.7	0.630

[a] Minor morph of *O. lyrataeformis*

[b] Minor morph of *O. kolchida*

♀♀ – female nematodes

*Statistically significant

n – number of infected individuals

Supplementary Table S4. Histopathological scoring of the moose abomasum.

Animal	Intensity of infection with gastrointestinal nematodes	Exfoliation of the mucosa epithelium	Inflammatory infiltrates in the mucosa	Inflammatory infiltrates in the submucosa	Tunica mucosa thickening and cavern formation	HP score sum
1.	3520	0	0	0	0	0
2.	18195	1	0	2	0	3
3.	77890	2	1	2	2	7
4.	57425	2	1	2	3	8
5.	98159	2	2	3	3	10
6.	40963	1	1	1	2	5
7.	6400	0	0	1	0	1
8.	9400	1	0	1	0	2
9.	40882	1	1	1	1	4
10.	23650	1	0	2	0	3
11.	14780	1	0	1	0	2
12.	43890	1	1	1	0	3
13.	97	0	0	0	0	0
14.	5280	0	0	1	1	2
15.	14035	1	0	1	1	3
16.	60	0	0	0	0	0

0 – no lesions; 1 –minimal; 2 – mild; 3 – moderate; 4 - severe

Supplementary Table S5. Histopathological scoring of the moose liver.

Animal	The intensity of *P. fasciolaemorpha* infection	Cirrhosis / liver fibrosis	Inflammatory infiltrates	Lobule architectural disturbance	Formation of connective tissue capsules	HP score sum
1.	11 150	4	3	2	4	13
2.	10 126	3	3	2	4	12
3.	2 128	2	3	1	3	9
4.	2 650	2	2	1	3	8
5.	9586	3	3	2	4	12
6.	189	0	0	0	1	1
7.	1 504	1	1	0	2	4
8.	188	0	1	0	1	2
9.	4	0	0	0	0	0
10.	0	0	0	0	1	1
11.	56	0	1	0	0	1
12.	300	1	2	0	2	5
13.	0	0	0	0	0	0
14.	268	1	2	0	1	4
15.	0	0	0	0	0	0
16.	0	0	0	0	0	0
17.	0	0	0	0	0	0
18.	0	0	0	0	0	0
19.	0	0	0	0	0	0
20.	96	1	1	0	0	2

0 – no lesions; 1 –minimal; 2 – mild; 3 – moderate; 4 - severe

Supplementary Table S6. Prevalence and infection intensity of eggs, oocysts and larvae of parasites in the feces according to the studied region of Poland. The arrows indicate how the prevalence and number of excreted eggs, oocysts and larvae differed.

PREVALENCE

Parasite	Central Poland (*n* = 101)		Eastern Poland (*n* = 148)		Northeastern Poland (*n* = 40)		
	No. of infected moose	Prevalence (CI 95%)	No. of infected moose	Prevalence (CI 95%)	No. of infected moose	Prevalence (CI 95%)	*p*
Trichostrongylidae	100	99.0 (94.6–99.8)	147	99.3 (96.3–99.9)	39	97.5 (87.1–99.6)	0.599
Nematodirella alcidis	49	48.5 (39.0–58.1)	108	73.0 (65.3–79.5)↑	20	50.0 (35.2–64.8)	<0.001*
Aonchotheca sp.	10	9.9 (5.5–17.3)	24	16.2 (11.1–23.0)	3	7.5 (2.6–19.9)	0.191
Trichuris spp.	72	71.3 (61.8–79.2)	104	70.3 (62.5–77.0)	23	57.5 (42.2–71.5)	0.244
Elaphostrongylus sp.	13	12.9 (7.7–20.8)	35	23.6 (17.5–31.1)	11	27.5 (16.1–42.8)	0.057
Varestrongylus sp.	6	5.9 (2.8–12.4)	20	13.5 (8.9–20.0)	2	5.0 (1.4–16.5)	0.078
Dictyocaulus sp.	2	2.0 (0.5–6.9)	3	2.0 (0.7–5.8)	1	2.5 (0.4–12.9)	0.979
Moniezia spp.	5	5.0 (2.1–11.1)	15	10.1 (6.2–16.0)	7	17.5 (8.7–32.0)	0.062
Parafasciolopsis fasciolaemorpha	60	59.4 (49.7–68.5)↔	118	79.7 (72.5–85.4)↑	8	20.0 (10.5–34.8)↓	<0.001*
Paramphistomidae	63	62.4 (52.6–71.2) ↑	22	14.9 (10.0–21.5) ↔	0	0 (0–8.8) ↓	<0.001*
Eimeria alces	3	3.0 (1.0–8.4)	6	4.1 (1.9–8.6)	1	2.5 (0.4, 12.9)	0.844
Eimeria catubrina	1	1.0 (0.2–5.4)	1	0.7 (0.1–3.7)	0	0 (0–8.8)	0.815

INTENSITY

Parasite	Central Poland		Eastern Poland		Northeastern Poland		
	No. of infected moose	Median (range)	No. of infected moose	Median (range)	No. of infected moose	Median (range)	*p*
Trichostrongylidae	100	9 (<1–1076)	147	8 (<1–503)	39	2 (<1–193)↓	0.001*
Nematodirella alcidis	49	1 (<1–7)	108	1 (<1–30)	20	1 (<1–11)	0.087
Aonchotheca sp.	10	<1 (<1–1)	24	<1 (<1–4)	3	<1, <1, 1	0.933
Trichuris spp.	72	6 (<1–379)	104	4 (<1–978)	23	2 (<1–94)↓	0.016*
Elaphostrongylus sp.	13	3 (<1–86)	35	7 (<1–175)	11	17 (<1–251)	0.368
Varestrongylus sp.	6	3 (<1–4)	20	1 (<1–15)	2	1, 2	0.192
Dictyocaulus sp.	2	1, 1	3	<1, 1, 1	1	<1	–
Moniezia spp.	5	27 (13–61)	15	18 (<1–217)	7	27 (1–40)	0.340
Parafasciolopsis fasciolaemorpha	60	1 (<1–82)↓	118	1 (<1–872)	8	1 (<1–3)	0.005*
Paramphistomidae	63	1 (<1–19)↑	22	1 (<1–6)	0	–	0.006*
Eimeria alces	3	<1, 1, 1	6	3 (1–8)	1	2	–
Eimeria catubrina	1	43	1	<1	0	–	–

*statistically significant

CI 95% – the upper and lower bounds of the confidence interval
